# Nanoremediation of tilapia fish culture using iron oxide nanoparticles biosynthesized by *Bacillus subtilis* and immobilized in a free-floating macroporous cryogel

**DOI:** 10.1186/s12917-024-04292-5

**Published:** 2024-10-09

**Authors:** Basma Sheta, Mohammed El-Zahed, Mona Nawareg, Zeinab Elkhiary, Salahuddin Sadek, Ayman Hyder

**Affiliations:** 1 Zoology departments, Faculty of Science, Damietta University, New Damietta, 34517 Egypt; 2 Botany & microbiology departments, Faculty of Science, Damietta University, New Damietta, 34517 Egypt

**Keywords:** Green iron oxide nanoparticles, Nano hydrogel, Water nano remediation, Nile tilapia (*Oreochromis niloticus*), Fish culture, Cadmium pollution

## Abstract

**Background and aim:**

Contamination from increased anthropogenic activities poses a threat to human health as well as the ecosystem. To develop a nanotechnological approach to improve aqua fisheries, we synthesized magnetic hematite nanoparticle-based gel and evaluated its efficacy in a cadmium-polluted closed system to decontaminate water and improve tilapia fish health.

**Methods:**

Green iron oxide nanoparticles were biosynthesized by the metabolite of *bacillus subtilis* and incorporated into polyvinyl alcohol to construct a hydrogel by cryogelation.

**Key findings:**

The cryogel had interconnected macropores with diameters widely ranging between 20 and 200 μm and could be free-floating in water. When applied in cadmium-polluted tilapia culture, this nanogel reduced turbidity and ammonia in the aquarium, adsorbed cadmium from the water with a larger quantity on the gel’s outer surface than in its center., and reduced cadmium concentration in tilapia’s liver, gills, and muscles. Application of this nano-based cryogel reduced the toxic effects of cadmium on tilapia fish. It maintained hepatic and renal cell nuclear integrity as determined by comet assay. This nano-treatment also reversed the cadmium-induced elevations of plasma lipids, glucose, stress marker cortisol, the hepatic enzymes AST and ALT, and the kidney function marker urea, and improved the lymphocytopenia and other hematological functions in tilapia fish intoxicated by cadmium.

**Supplementary Information:**

The online version contains supplementary material available at 10.1186/s12917-024-04292-5.

## Significance

The nano product of this work has many advantages. It is cheap, green, safe, can be easily manufactured, replaced, or removed from the aquaria, and proven efficient to clean aqua fisheries’ water. The concentration of NPs can be adjusted in the hydrogel, and the utilization of magnetic α-Fe_2_O_3_ NPs allows for the removal of the released particles by application of a magnetic field.

**Supplementary Information:**

The online version contains supplementary material available at 10.1186/s12917-024-04292-5.

## Introduction

Human industrial and urban activities and poor environmental monitoring lead to contamination of waterbodies with non-degradable heavy metals. Unprecedented concentrations of these heavy metals have been recorded in aquatic ecosystems [1], increasing their adsorption into sediments or their bioaccumulation in aquatic species. The resulted toxicity has an important consequence of changing the biodiversity and the entire aquatic ecosystem [2]. Nevertheless, consumption of heavy metal-contaminated fish threatens human health, including causing carcinogenicity and deterioration of many tissues and systems [3]. As an example of those heavy metals, cadmium is one of the most known environmental pollutants and biotoxicants that may harm human health. The concentration of cadmium in freshwater was reported to range from 10 to 4000 ng/L, while it reaches as high as 100 µg/L in polluted areas and even 1 mg/L in artificial wetlands [4]. Other anthropogenic sources of Cd include fertilizer use containing Cd, mining, and combustion emissions that are dumped into the atmosphere [5]. Cadmium contamination was reported to cause several deleterious effects including, among others, liver failure, kidney dysfunction, cardiovascular problems, metabolic disorders, neurotoxic and carcinogenic effects, and reproductive and respiratory system defects [6] in several organisms. To avoid all of this, chemical discharge into receiving waterbodies should be prevented and sustainable water treatment technologies are urgently needed [7].

To remove toxic heavy metals from culture and waste waters, many technologies ranging from simple to complex have been developed. Variable physicochemical methods including precipitation, filtration, ion exchange, reverse osmosis, and adsorption, are commonly applied [8, 9]. However, physicochemical techniques are expensive and work only in the presence of high concentrations of heavy metals in the treated water [10]. In fact, most of these techniques were developed in the early 20th century and are not sufficient to face the challenges of the present pollution problems [11]. Bioremediation techniques, including phytoremediation, bio-sorption (treatment with dead biomass), bio-reduction, and intracellular bioaccumulation (by specific organism) have been applied, and are reasonable and ecofriendly [12] and phytoremediation was proven to be effective in removing contaminants from tilapia fish culture [7]. However, special extra care should be given to the added organisms as, for example, added plants, bacteria, fungi, and algae require separate areas to grow, extra handworks, extra times, and can cause changes in the ecosystem, if heavily applied. In addition, the biosorption action is not as powerful, when compared to that of other means such as adsorption by nanoparticles [13].

Nanotechnology is an interesting emerging innovative alternative for aquaculture and wastewater treatment. Nanoparticles are reported to have a great reactivity and adsorption properties for heavy metals due to, among many reasons, their nano size, huge surface area, and increased tensile strength. Meanwhile, they are light, cheap, easy to prepare, effective, energy independent, and ecofriendly systems [14, 15]. However, most research are interested in the adsorptive efficiency of nanoparticles and their ability to clean water [13, 16], but not the biological effects on aquatic organisms, although nanoparticles themselves have proven their own toxicity even to fetuses after passing through placenta [17]. The recovery of NPs from water is problematic, and the loss of nanoparticles in waterbodies may result in other environmental complications [11]. In this context, accumulated higher concentrations of silver nanoparticles than 30 µg/L were suggested to have potential toxic effects on tilapia [18]. It is therefore essential to immobilize NPs in a structure that allows for heavy metal adsorption but prevents the direct toxicity of NPs. In this context, three-dimensional polymer networks known as hydrogels can be synthesized using either physical or chemical cross-linking techniques. Because they are highly biocompatible with biological tissues, hydrogels are useful in biomedicine, tissue engineering, and the treatment of wastewater [19, 20]. As a biocompatible, non-toxic, and safe for human use, polyvinyl alcohol (PVA) could be treated readily using physiochemical methods to produce hydrogels [21].

In the present work, we applied a green nano system consisting of a free-floating macroporous cryogel immobilizing biosynthesized nanoparticles to adsorb the contaminating cadmium in tilapia fish (*Oreochromis niloticus*) culture. The aim of the gel was to prevent the direct contact between the fish and nanoparticles, and the consequent possible nanoparticle-induced fish toxicity. Tilapia was recruited because it is one of the most internationally consumed aquatic organisms due to their elevated proteins, lipids, minerals and other essential nutrients [22, 23]. As well, tilapia was reported as a bioindicator of water pollution, to monitor the water quality and to study the biological influence of heavy metals and the mechanisms of biological adaptations [24]. This applied nano system in the present study is introducing an efficient, costly effective, ecofriendly, and novel approach for a successful remediation of heavy metals in fish culture water.

## Materials and methods

### Preparation of microbial cell-free metabolites

Five tested microbial strains including the bacteria (*Bacillus subtilis* ATCC 6633, *Escherichia coli* ATCC 25922, *Raoultella ornithinolytica* ATCC 31898, and *Leclercia adecarboxylata* ATCC 23375) and the yeast *Saccharomyces cerevisiae* ATCC 9763 were sub-cultured on nutrient agar (Oxoid, UK) and yeast extract peptone agar (YEPA, Oxoid, UK) plates. A standard 0.5 MacFarland (1–2 × 108 CFU/ml) from each strain was prepared, inoculated into fresh sterile broth medium and incubated at 37 °C in a shaking incubator at 37 °C/120 rpm (LSI-3016R, Daihan Lab Tech, South Korea) for 48 h. After the incubation period, the microbial culture suspensions were centrifuged for 20 min at 8000 rpm and then filtered using a 0.2-µm surfactant-free cellulose acetate Nalgene syringe filter (Thermo Scientific Inc., USA) to separate the microbial biomass from the supernatant.

### Green synthesis of iron oxide nanoparticles by bacterial metabolite

Fe_2_O_3_ NPs were synthesized according to the method described by Sundaram et al. [25]. In 500 ml Erlenmeyer flasks, cell-free supernatant of each microbial strain was resuspended in 100 ml of 5 mM FeCl_3_.6H2O (Sigma-Aldrich, USA) aqueous solution at ratio of 1:1 (v/v%) and stirred at 200 rpm at room temperature (25 °C) in the presence of sunlight. The reaction was conducted until the color changed from pure golden yellow to turbid brown as an indication for the biosynthesis of Fe_2_O_3_ NPs. Ultraviolet-Visible Spectroscopy (UV-Vis) in the range 200–600 nm was done using UV-Vis spectrophotometer V-760 (JASCO, UK) for the preliminary determination of Fe_2_O_3_ NPs [25]. The highest concentration Fe_2_O_3_ NPs-producing strain was selected for the biosynthesis of Fe_2_O_3_ NPs.

### Characterization of nanoparticles

UV-Vis spectrum of Fe_2_O_3_ NPs was studied using UV/VIS/NIR Spectrophotometer V-630, Japan (Central Lab, Faculty of science, Damietta University, Egypt). Transmission electron microscopy (TEM) images were investigated using JEOL JEM-2100, Japan and zeta potential by using the Zeta sizer instrument (Malvern Instruments Ltd; zs90, Worcestershire, UK) at TEM Unit, Mansoura University, Egypt. X-ray diffraction (XRD) results were taken using the model LabX XRD-6000, Shimadzu, Japan (Nanotechnology Center, Kafrelsheikh University, Egypt). Fourier transform infrared spectroscopy (FTIR) spectra were obtained by using the SpectrumTwo IR Perkin Elmer instrument. FTIR spectrum of Fe_2_O_3_ NPs was recorded by FT/IR-4100typeA (Central Lab, Faculty of science, Damietta University, Egypt).

### Preparation of PVA/Fe2O3 NPs hydrogels

Polyvinyl alcohol (PVA)-aldehyde macroporous hydrogel was prepared with the method previously described [19, 26], with modifications necessary to combine the gel with the nanoparticles. Briefly, PVA solutions were synthesized by combining 20 g of PVA with 200 ml Millipore water and stirred magnetically at 90 °C. After complete dissolution, different concentrations of sonicated Fe_2_O_3_ NPs (0.1–0.5 mg/ml) were mixed with the PVA solutions and stirred for 10 min. After cooling to 55 °C, aliquots 0.3 ml of glutaraldehyde solution (50%) was gradually added to the previous mixture and immediately poured into glass dishes (200 mm) with thickness of 5 mm and frozen at -18 °C for 20 h. Cryogelation of PVA/Fe_2_O_3_ NPs hydrogels was done using three successive frost (20 h at -18 °C) and defrost (20 h at room temperature) cycles. After cryogelation, hydrogels were dialyzed for two weeks against 5 l of deionized water to remove any unreacted glutaraldehyde.

### Antibacterial activity of PVA/Fe2O3 NPs hydrogels

The antibacterial action of PVA/Fe_2_O_3_ NPs hydrogels was tested against Gram-positive bacteria (*B. subtilis* and *Staphylococcus aureus*) and Gram-negative bacteria (*E. coli* and *Pseudomonas aeruginosa*) using agar well diffusion method [27]. 5 mm discs from different PVA/Fe_2_O_3_ NPs hydrogels were prepared and tested using Mueller Hinton Agar (MHA) plates inoculated by 0.5 McFarland of the tested bacteria. After incubation at 37 °C for 48 h, inhibition zones were measured and recorded in mm.

### Fish and treatment grouping

Animal experiments comply with the ARRIVE guidelines and were carried out in accordance with the U.K. Animals (Scientific Procedures) Act, 1986 and associated guidelines, and EU Directive 2010/63/EU for animal experiments. The institutional ethical committee for animal research authorized and approved all the procedures and animal handling (Approval number and date: DuREC No 13 on Feb 27, 2023). Juveniles of tilapia fish (*Oreochromis niloticus*) with a mean weight 18.64 ± 0.54 g and mean length 10.34 ± 0.21 cm were purchased from local fish farms and left for two weeks for acclimatization to laboratory conditions prior to experimentation as described elsewhere [28]. The fish were fed twice daily (9:00 AM − 4:00 PM) at 3% of their body weight with commercial feed containing 25% crude protein. Glass aquaria with dimensions of 70 × 30 × 20 cm and a capacity of 42 l of dechlorinated tap water were used in this study. They were divided into 3 groups; 2 of them contained 2 mg/l [29] of cadmium chloride (CdCl_2_), while the 3rd was assigned to the control group without cadmium. After acclimatization, 15 tilapia fishes were transferred to each tank. One of the 2 cadmium groups was provided with the hydrogel immobilized with 0.1 mg/ml Fe_2_O_3_ NPs. *Oreochromis niloticus* in all aquaria were exposed to their specific treatments for 14 days. The water was changed twice a week to maintain the rearing conditions and CdCl_2_ concentrations. Water temperature, pH, dissolved oxygen, Ammonia and turbidity were measured twice weekly. Water temperature and pH were measured by (AD11-PH Meter), Dissolved oxygen was measured by (AD610- DO Meter), Ammonia was measured by (HI715 - handheld colorimeter Ammonia MR) and turbidity was measured by (Turbidity meter TU-2016 Lutron, Taiwan).

### Estimation of cadmium concentration in fish tissue and nanogel

Following the experiment, three fish from each aquarium were euthanized with an overdose of buffered MS-222 (tricaine methanesulfonate, 200 mg/L) in a separate aquarium. These fish had their liver, muscles, and gills removed to estimate the amount of cadmium. Three nanogels were separated into sections for the same reason. Gel samples were taken from the gel’s core, periphery, and sub-periphery. Following a 48-hour oven drying process at 80 °C, all samples were digested using concentrated nitric acid (69%) and perchloric acid (70%) in a 2:1 ratio. The digestion was kept until the solution turned transparent. An atomic absorption spectrometer (PinAAcle 500, Perkin Elmer) was used to measure the amount of cadmium present in the filtered solution. Atomic absorption spectrophotometry was performed at the Water Research Microanalysis Laboratory, Damietta University. Cadmium was measured at 228.8 nm with a hollow cathode lamp. A standard curve was done using standard solutions of 0.005, 0.01, 0.05, 0.25, 2.5, and 10 ppm. Cadmium concentration was expressed as µg/g dry weight.

### Fish body composition

For body chemical composition analysis, a tilapia fish sample was obtained from each treatment. Moisture, crude proteins and lipids of the entire tilapia body were estimated on a dry matter basis. By weighing a sample that had been previously weighed and dried at 105^o^C for at least 12 h, until total dryness. The sample’s water content was determined by the difference between the beginning and final weights. Crude protein was calculated by multiplying total nitrogen content estimated by the semi-automatic Kjeldahl method by 6.25, and crude lipids was estimated by extraction in a Soxhlet device using petroleum ether as an extraction solvent [30]. Body composition analysis was performed in the Water Research Microanalysis Laboratory at the Faculty of Science, Damietta University, Egypt.

### Hematological and biochemical analyses

Blood samples were collected from the caudal vein of euthanized fish according to Feldman et al. [31]. A complete blood count, hemoglobin content, and hematocrit value were measured directly. Plasma samples were gained by blood centrifugation (4000 g for 10 min at 4 °C). plasma glucose (mg/l), total protein (g/dl), total lipid (g/l), urea (mg/dl), the activity levels of aspartate aminotransferase (AST) and alanine aminotransferase (ALT), and cortisol were determined with commercial kits from Reactivos GPL (CHEMELEX, Barcelona, Spain).

### Comet assay

In tilapia livers and kidneys of various groups, single cell gel electrophoresis (Alkaline Comet test) (Olive and Banáth, 2006) [32] was used to examine and measure cellular DNA damage in response to cadmium toxicity and the nano-treatment. After experimental tilapia cultures, small fragments of both tissues were carefully ground and10 µl of the suspension and 70 µl of 0.5% low-melting agarose were combined. A slide covered with 1% normal melting agarose was used to immobilize the combination. Dried slides were first incubated in cold lysis buffer at 4 °C for 24 h in the dark prior to being incubated in new alkaline electrophoresis buffer and neutralized with Tris buffer. Prepared slides were electrophoresed for 20 min at 25 V and 300 mA. Finally, slides were air-dried, fixed in 100% cold ethanol, stained with propidium iodide (PI, Merck), examined using a Zeiss Axioplan epifluorescence microscope, and comet score was recorded in fifty comet nuclei per slide.

### Statistical analysis

Results are expressed as mean ± standard error of means (SEM). One-way analysis of variance (ANOVA) followed by t-test as a post-hoc was used to estimate the statistical difference between the 3 groups. Statistical analysis was performed using Microsoft Excel. A *P* value < 0.05 was considered to be significant.

## Results

### Characterization of iron oxide NPs

**Fig. 1:**
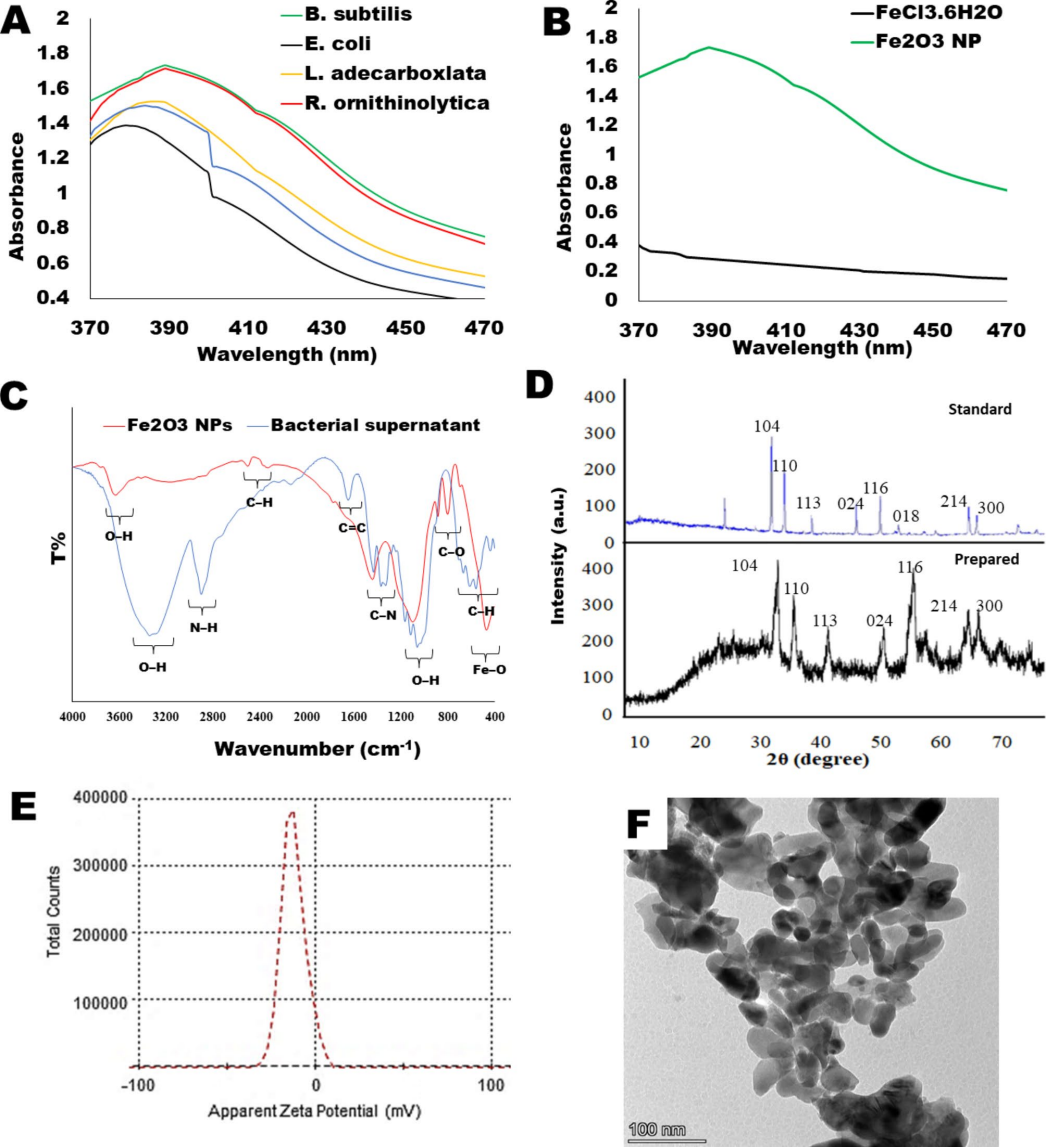
The green-synthesized iron oxide nanoparticles: **(A)** UV-Visible spectroscopy of Fe_2_O_3_ nanoparticles biosynthesis using different microbial metabolites. **(B)** Difference of absorbance in spectroscopy between iron oxide nanoparticles and ferrous chloride. **(C)** Fourier transform infrared spectroscopy (FTIR) spectra analysis of iron oxide nanoparticles. **(D)** X-ray diffraction (XRD) analysis of iron oxide nanoparticles. **(E)** Zeta potential analysis of iron oxide nanoparticles, showing a sharp peak at -21.1 mV. **(F)** Transmission electron microscopy (TEM) analysis of the green iron oxide nanoparticles

A color change from pure golden yellow of FeCl_3_.6H_2_O solution to turbid brown occurred within 15–20 min as a first sign for the successful formation of Fe_2_O_3_ NPs. The biosynthesis process of NPs was confirmed by UV-Vis Spectroscopy, FTIR and XRD spectra. An absorbance peak at 390 nm was observed in the UV-Vis spectrum of Fe_2_O_3_ NPs which indicated to an excitation wavelength of α-Fe_2_O_3_ NPs band as described by Shikha et al. [33]. Figure 1A shows that all tested microbial strains had the ability to biosynthesized Fe_2_O_3_ NPs in the presence of sunlight. Fe_2_O_3_ NPs revealed a broad peak at 391 nm, which agree with previous findings [34, 35]. Among all microbes, the *B. subtilis* strain was selected for the extracellular biosynthesis of Fe_2_O_3_ NPs in a high concentration and within 15 min. The formation of Fe_2_O_3_ NPs was confirmed using UV–vis spectroscopy analysis (1B), as compared with the spectra of ferric chloride.

In the FTIR results, Fe_2_O_3_ NPs (Fig. 1C) showed peaks at 3617 cm^− 1^ resembling to polyphenols (O–H), 2494 and 2327 cm^− 1^ assigned to C–H bond of alkenes, 1430 cm^− 1^ corresponding to nitro group (N–O), 1058 cm^− 1^ resembling to C–C, 878 cm^− 1^ assigned to C–O bond. Stretching vibrations of metal-oxygen bonds appeared at the FTIR spectrum of Fe_2_O_3_ NPs between 400 and 700 cm^− 1^ (at 458 cm^− 1^), confirming the presence of Fe-O bond and Fe-O-Fe stretching vibration [36, 37].

The XRD spectrum of Fe_2_O_3_ NPs (Fig. 1D) shows intense diffraction peaks at 33.6°, 36.1°, 41.4°, 49.9°, 54.3°, 62.8° and 64.6° resemble to (104), (110), (113), (024), (116), (214) and (300) crystallographic planes of inverse spinel magnetite phase. Crystallographic lattice planes of (104), (113) conforming the presence of traces α-Fe_2_O_3_ NPs hematite phase at diffraction angles at 33.6° and 41.4°, which match with Joint Committee on Powder Diffraction Standards (JCPDS) card number 33–0664 (ICDD file number: 13–534) [38, 39]. Using the Scherrer equation d = kλ/βcosθ [40], where k is the Scherrer constant (0.54), λ is the x-ray wavelength (1.54 A°), β is the half width of the peak, and *θ* is the Bragg’s angle, it was determined that the average crystalline size was 38 nm.

The Zeta potential of Fe_2_O_3_ NPs was − 21.1 mV (Fig. 1D), indicating moderately stable NPs.

The morphology and size of the Fe_2_O_3_ NPs were examined by TEM. TEM images showed polygonal and quasi-spherical-shaped Fe_2_O_3_ NPs with an average size of 20–40 nm (Fig. 1E).

Taken together, these results confirm the biosynthesis of α-Fe_2_O_3_ NPs using the metabolite of *B. subtilis* at room temperature. The characterization data agree with some previously reported results [41].

### Application of the nano-based gel in tilapia culture

**Fig. 2:**
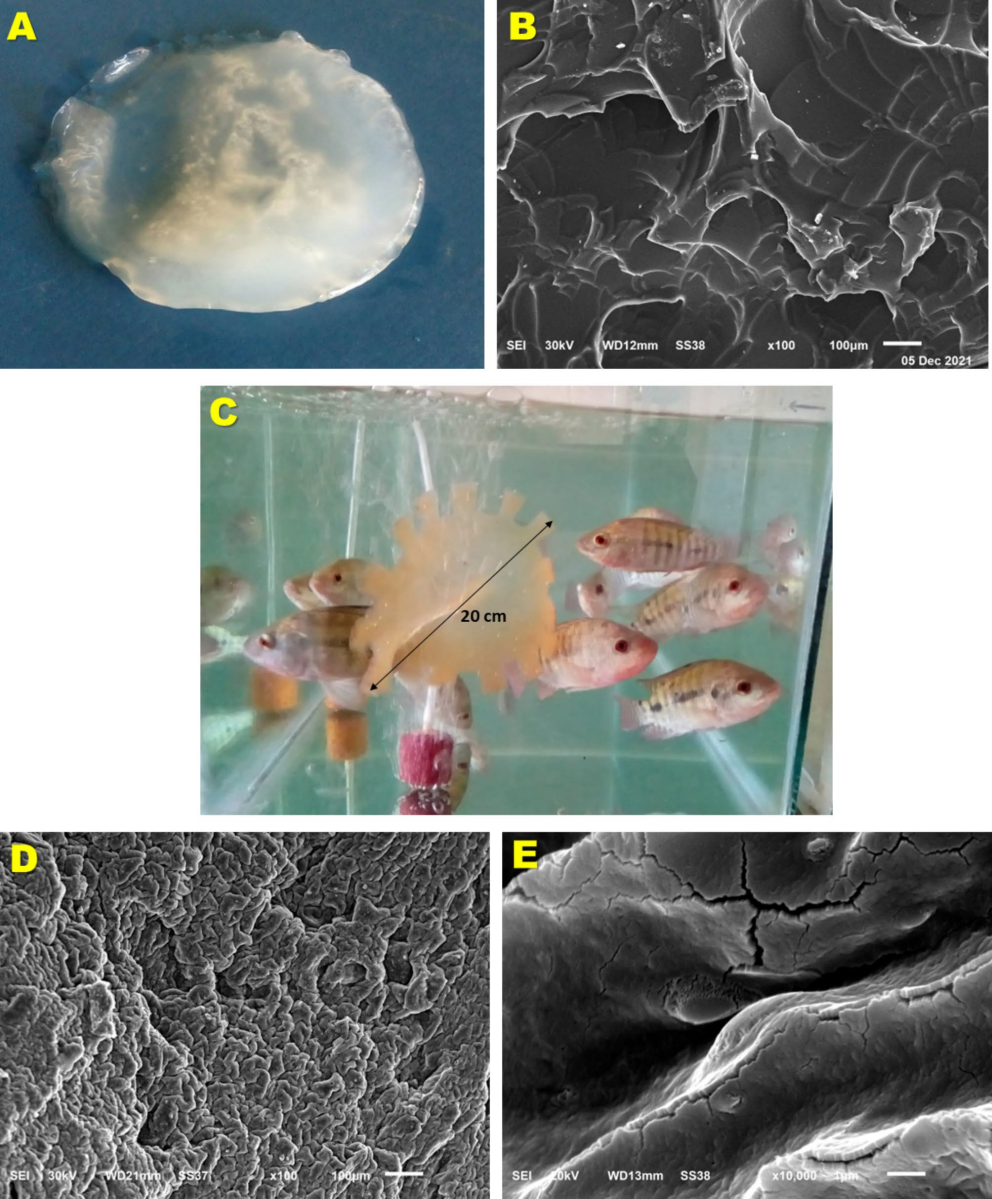
The polyvinyl alcohol (PVA) hydrogel: The PVA-aldehyde hydrogel without immobilized NPs (**A**) is prepared by cryogelation.as described in the material and methods section. **B)** Scanning electron microscopy of the prepared macroporous gel (scale bar 100 μm). **C)** The biosynthesized iron oxide–containing hydrogel is free-floating in the tilapia aquarium. A video is available in the supplementary data. **D)** Structure of the gel at the end of culture period (scale bar 100 μm). Compare with subfigure B. **E)** A magnification of D showing the gel surface covered with a layer of amorphous material (scale bar 1 μm)

The polyvinyl alcohol – glutaraldehyde cryogel presented in this study looked like sponge (Fig. 2A) with interconnected macropores that resulted from the freezing of the reactants. It was not the aim of the present study to physiochemically characterize the prepared PVA-GA cryogel or to analyze its pore structure and size or the adsorption kinetics and capacity, since these analyses were conducted several times in previous works [42–46]. However, it was clear, as shown in Fig. 2B, that the gel was thin-walled and extremely porous with clear observable microchannels. The pores/channels were heterogenous in their structure with diameters widely ranged between 20 and 200 μm or even greater.

Figure 2C, and the **video** in the supplementary files, show the thin free-floating Fe_2_O_3_ NPs-immobilized cryogel. The gel was light red, which is the dominant color of the Fe_2_O_3_ NPs. After a period of floating, the applied gel became heavier due to water absorption and accumulation of biomaterial. At the end of the culture period, the scanning electron microscopic analysis (Fig. 2D) showed that the porosity and microstructure of the hydrogels was not preserved. Pores became narrower or even closed. Walls became opaque, thick and rough, as compared with the structure of the newly prepared gel (Fig. 2B). Figure 2E shows that amorphous material covers the walls of the gel, changing its shape and concealing its pores.

### Antibacterial activity of PVA/Fe2O3 NPs hydrogels

The antibacterial activity of PVA/Fe_2_O_3_ NPs hydrogels against *B. subtilis*, *S. aureus*, *E. coli* and *P. aeruginosa* was studied using the agar well diffusion method as shown in Table 1. The 0.1 mg/ml PVA/Fe_2_O_3_ NPs hydrogel has the ability to inhibit all tested microbes with inhibition zones of 20 and 17 mm against G-ve *E. coli* and *P. aeruginosa*, respectively, as well as 18 and 16 mm against G + ve *B. subtilis* and *S. aureus*. High concentrations from Fe_2_O_3_ NPs did not produce notable increasing in the antibacterial activity, which may refer to the low releasability of NPs from the hydrogel (El-Zahed et al., 2021). Overall, it was noted that PVA/Fe_2_O_3_ NPs hydrogel was more effective against G-ve bacteria than G + ve bacteria. This action might be due to the G + ve bacterium’s strong cell wall, which is made up of thick layers of peptidoglycan and so prevents the entry of Fe_2_O_3_ NPs into the bacterial cell [47].

**Table 1:** Agar well diffusion method test of PVA/Fe_2_O_3_ NPs

PVA/Fe_2_O_3_ NPs hydrogel	Inhibition zone diameters (mm ± SD)			
	B. subtilis	S. aureus	E. coli	*P*. aeruginosa
0.1^*^	18 ± 0.03	16 ± 0.03	20 ± 0.03	17 ± 0.06
0.2	20 ± 0.14	18 ± 0.03	23 ± 0	18 ± 0.06
0.3	21 ± 0.06	18 ± 0.06	24 ± 0	18 ± 0.03
0.4	22 ± 0	19 ± 0.14	24 ± 0	18 ± 0.06
0.5	22 ± 0	19 ± 0	25 ± 0	18 ± 0.03

^*^ The concentration of Fe_2_O_3_ NPs (mg/ml) in PVA/Fe_2_O_3_ NPs hydrogel

### The prepared hydrogel adsorbs cadmium

**Fig. 3:**
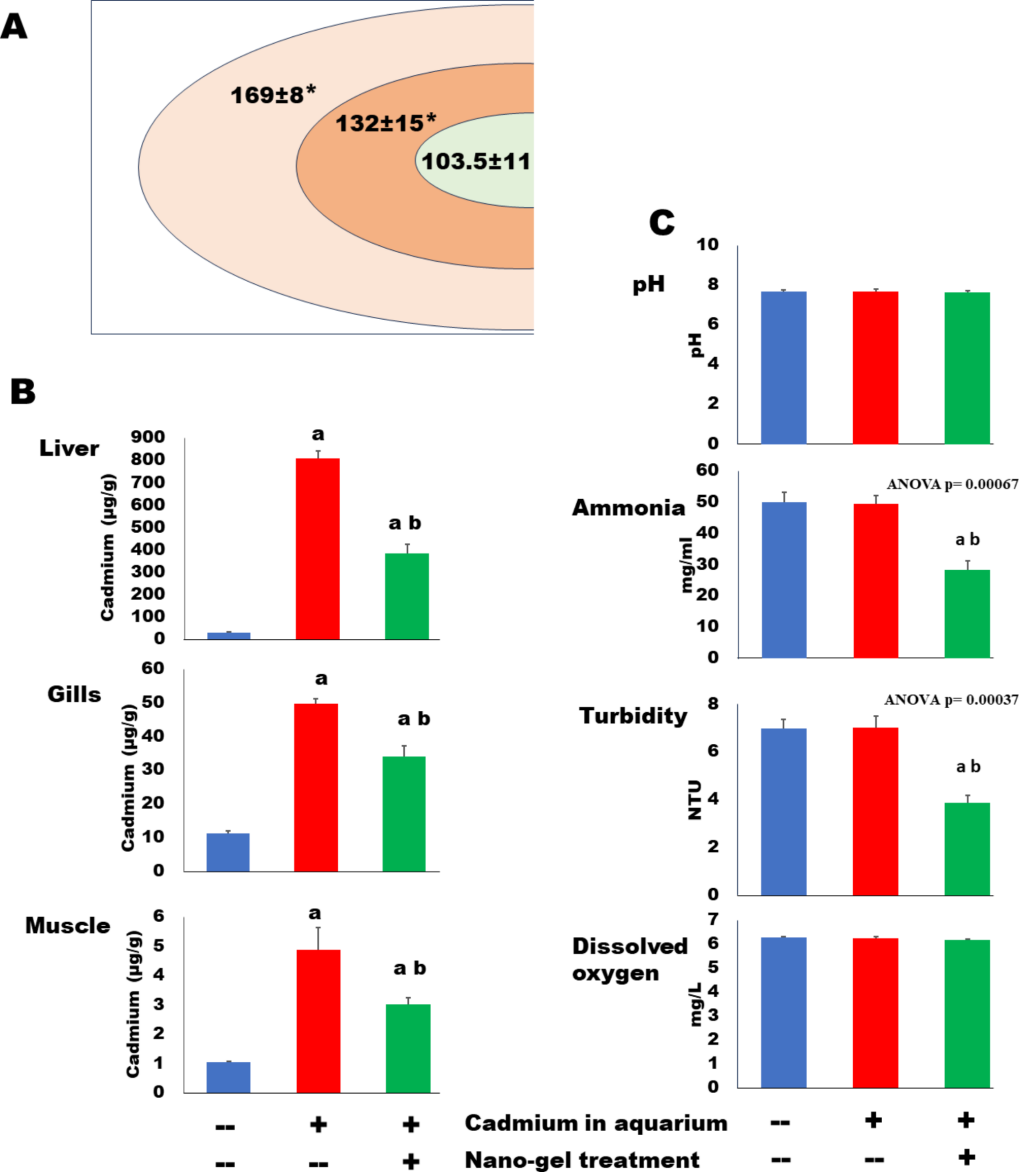
Iron oxide nanoparticles immobilized in PVA hydrogel adsorb cadmium and clean water in tilapia fish culture. **(A)** Cadmium concentration (ug/ml) in the gel. The gel was divided vertically into 3 pieces from the outer surface to the core in the same area. Data are shown as mean ± SEM of *N* = 3. The “*” denotes that all values are significantly different from each other (t-test after ANOVA) and show a concentration gradient from the surface to the core. **(B)** The adsorption of the heavy metal by the nano treatment of Cd-polluted tilapia culture significantly reduces cadmium concentration (ug/g dry wt) in different fish tissues. *N* = 3. Statistical analyses: ANOVA < 0.05 for all organs. “a” denotes higher Cd concentration than the control value, and “b” denotes a significantly lower value than that of the Cd only – treated group (t-test as a post-hoc test). **(C)** Effect of iron oxide nanoparticles immobilized in PVA hydrogel on Cd-polluted tilapia fish culture water physicochemical characters. Data are mean ± SEM values of samples collected twice weekly. ANOVA value was mentioned if significant. Posthoc t-test: “a” and “b” denote lower value than those of control and Cd-treated groups, respectively

Cadmium concentration was estimated in the cryogel immobilizing Fe_2_O_3_ NPsafter 14 days in aquaria of tilapia fish culture. The results revealed a concentration gradient in the gel from the outer surface to the core (Fig. 3A). This adsorption led to decreasing cadmium concentration in the cultured fish tissues. This concentration was estimated in 3 tilapia organs: liver, muscle, and gills (Fig. 3B). Cadmium was significantly decreased in these tissues in the cadmium-nano-treated group as compared with the cadmium only treated group. In addition, characters of culture water were also improved by the nano-gel. Treatment with this nano-gel significantly decreased turbidity and ammonia content not only in comparison with the cadmium-treated culture, but also with the control culture [Fig. 3C].

### Nano treatment maintains the nuclear integrity of cadmium-intoxicated tilapia

**Fig. 4:**
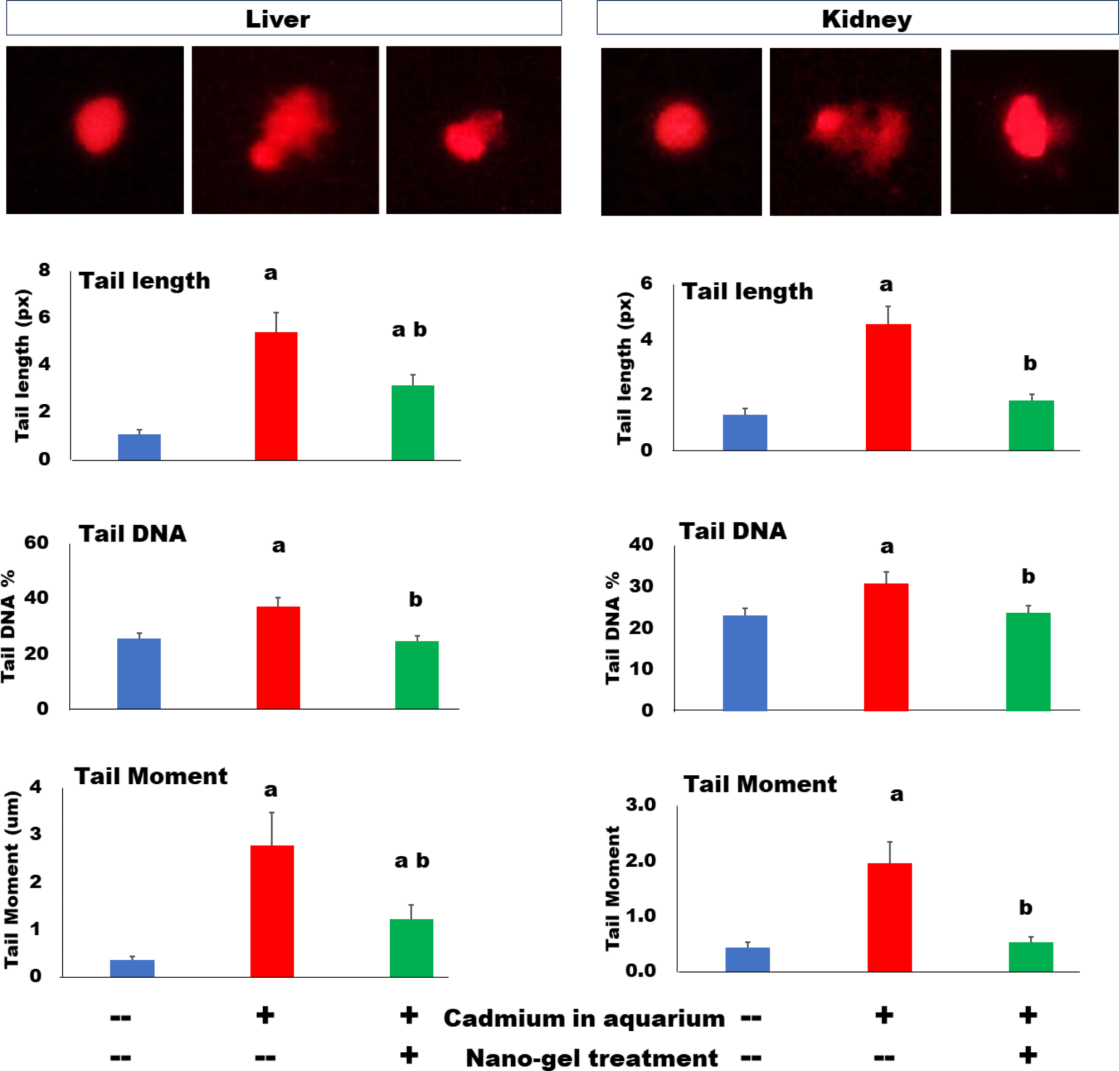
Reduction of Cd-induced DNA damage in liver (left panel) and kidney (right panel) of tilapia fish by iron oxide nanoparticles immobilized in PVA hydrogel. Single cell DNA damage was visualized by comet assay. The upper images represent the comet results of the control, cadmium-treated, and Cd-nanocomposite-treated groups, respectively. Data of comet analysis are mean ± SEM values of samples collected at the end of the tilapia culture period. ANOVA value was significant (*P* < 0.05) for all tested parameters. Posthoc t-test: “a” denotes significantly higher value than the control value, and “b” denotes a significantly lower value than that of the Cd–treated group

Significant DNA damage was recorded in both livers and kidneys of tilapia fish exposed to cadmium toxicity for 14 days (Fig. 4). DNA integrity defects were proved by comet assay, which results in different parameters that can be quantified. Thus, the results revealed significant increases in both the comet tail length (fragmented DNA), tail DNA quantity, and tail moment (= tail length x % tail DNA) in both liver and kidney cells. The adsorptive iron oxide nano treatment completely maintained the nuclear integrity in kidney, since similar quantitative results were obtained to the control results. This maintenance was only partial in the hepatic cells.

### Nano treatment reverses affected tilapia physiological aspects in cadmium-intoxicated culture

**Fig. 5:**
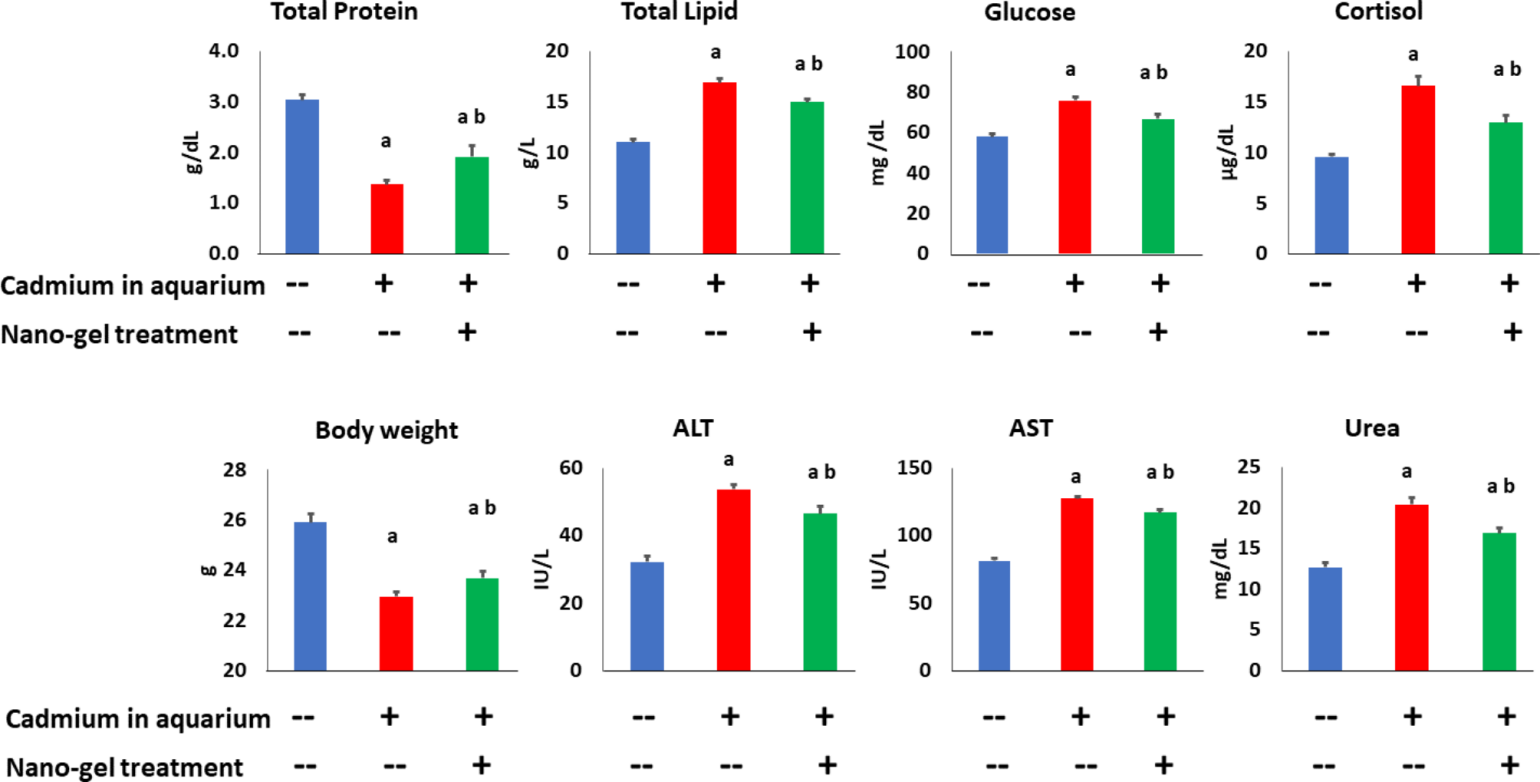
Nutritional and functional aspects of tilapia fish exposed to cadmium in culture and treated with hydrogel immobilizing iron oxide nanoparticles to adsorb the heavy metal. Tilapia was cultured in these conditions for 2 weeks. Data are presented as mean ± SEM of *N* = 3. ANOVA was significant (*P* < 0.05) in all measurements. “a” and “b” denote a significantly different value from that of control and cadmium-treated groups, respectively (t-test, *P* < 0.05)

As shown in Fig. 5, the toxic effect of 14-day-cadmium treatment on blood parameters of tilapia fish was obvious. Treatment with cadmium increased significantly both total plasma lipids, glucose, cortisol, the hepatic enzymes AST and ALT, and the kidney function marker urea, whereas it decreased significantly both plasma proteins and the fish body weight. Adsorption of cadmium by the iron oxide-gel-nano treatment reversed these effects significantly, when compared with the cadmium-treated cultures. However, the results of all of these parameters reveal that this restoration was only partial, since all values were still significantly different from the control values (Fig. 5).

The body composition was not largely altered by different treatments, since no significant differences were observed between different groups. The most obvious result was the non-significant increase of crude lipid content in cadmium-treated group (6.88%), compared to that of the control group (5.58%), and the cadmium-nano-treated group (6.42%). The crude protein was non-significantly decreased from 21.03% in the control group to 20.31% in cadmium-treated group. The nano treatment did not alter this content (20.42%). Similar results were obtained for fish body moisture (69.43, 68.12, and 70% for control, Cd-, and Cd-nano-treated groups, respectively.) and body ash (3.52%, 3.13% and 3.49% for control, Cd-, and Cd-nano-treated groups, respectively).

Hematological analyses in the present work are summarized in Table 2. In most parameters, blood parameters were harmed by exposure to cadmium, an effect that was partially lessened by the nanocomposite treatment. White blood cell (WBC) count in cadmium group was found to be significantly less than the control. This decrease is mostly due to the significantly reduced lymphocyte count. Noticeably, monocytes and neutrophils were doubled in the nano-treated group, when compared even with the control group. Cadmium exposure significantly decreased the RBC count, hematocrit value and hemoglobin concentration in tilapia fish. This hematopoietic impairment triggered by cadmium toxicity could be corrected by the nano treatment.

**Table 2:** Biochemical and hematological parameters of tilapia fish cultured in cadmium-polluted aquaria and treated with a hydrogel immobilizing green iron oxide nanoparticles

		Control	Cadmium	Cadmium + iron oxide Nanocomposite	ANOVA *p*
**Hb (g/dL)**		7.53 ± 0.57	4.50 ± 0.60 ^**a**^	6.33 ± 0.53	0.023
**RBCs x10** ^**6**^ **/µL**		1.36 ± 0.11	1.11 ± 0.16 ^a^	1.27 ± 0.22	ns (> 0.05)
**Hct %**		22.63 ± 1.23	14.30 ± 2.46 ^**a**^	19.87 ± 2.72 ^**b**^	0.0381
**MCV fL/cell**		167.67 ± 7.31	128.10 ± 6.07^**a**^	159.07 ± 11.91^**b**^	0.0394
**MCH pg/cell**		55.73 ± 1.86	40.70 ± 0.97^**a**^	51.53 ± 5.66	0.0444
**MCHC g/dl**		33.20 ± 0.74	31.83 ± 1.20	32.30 ± 1.61	ns (> 0.05)
**Platelets x10³/ µL**		29.22 ± 1.74	12.20 ± 1.20^**a**^	21.17 ± 1.59 ^**a, b**^	0.0006
**WBCs x 10³/µL**		59.77 ± 2.74	24.80 ± 2.50^**a**^	42.73 ± 1.97 ^**a, b**^	0.00015
	**Neutrophils x10³/µL**	1.56 ± 0.24	1.16 ± 0.48	5.17 ± 0.43 ^**a, b**^	0.0006
	**Lymphocytes x10³/µL**	53.93 ± 2.43	21.47 ± 1.79^**a**^	27.90 ± 1.41 ^**a, b**^	0.00004
	**Monocytes x10³/µL**	4.27 ± 0.24	1.73 ± 0.27^**a**^	7.63 ± 0.24 ^**a, b**^	0.00000
	**Eosinophils x10³/µL**	0.00 ± 0.00	0.43 ± 0.03^**a**^	2.03 ± 0.03 ^**a, b**^	0.00000
	**Basophils x10³/µL**	0.00 ± 0.00	0.00 ± 0.00	0.00 ± 0.00	ns (> 0.05)

Data are presented as mean ± SEM of *N* = 3. “a” and “b” denote significantly different values from control and Cd groups, respectively

## Discussion

The efficacy of fish culture water purification methods is of great direct concern since pollution is increasing as a result of growing anthropogenic activities. Of special interest, heavy metals are not biodegradable and accumulate in different organisms, with the consequence of being toxic and even carcinogenetic to human [48, 49]. Numerous articles demonstrated the high adsorptive capacity of nanoparticles for heavy metal contamination from aquatic solutions [13, 50–52]. However, fish long directly exposed to higher levels of nanoparticles exhibited variable intoxication responses, including inflammation, immune suppression, metabolic stress, biochemical disturbance, and growth retardation. These toxic effects depend on the exposure duration to NPs, and NP concentrations and size [53–55]. Similarly, non-magnetic nanoparticles have restricted applicability to purify water, since they are difficult to be separated from water [51]. This separation is crucial, since accumulation of NPs may be toxic per se. To isolate nanoparticles from the direct contact with the fish, preventing their toxicity, we thought to immobilize these NPs in a polymer structure (the hydrogel) that is free-floating like a jelly fish and can be easily removed or replaced. To reduce costs, cheap material such as ferrous sulphate and PVA have been used in synthesizing the nanoproduct. Nanoparticles were synthesized by a natural metabolite of *Bacillus subtilis*, implying the cost-effectiveness and usefulness of natural products [56].

The study focused primarily on water treatment using immobilized Fe_2_O_3_ NPs and studying the effect on tilapia fish health. Fe_2_O_3_ NPs have been chosen because they have many advantages, including the easy and cheap preparation applied method shown in the present study, reported adsorption efficiency for different heavy metals, and the ease of removal from water. The applied gel can be easily withdrawn from water. In addition, if there were magnetic α-Fe_2_O_3_ NP, the mean constituent in the gel, released in the culture water, they could be easily removed by application of a magnet or any magnetic field. The green preparation of NPs is also advantageous. It is easy, cheap, safe, and does not require any specific factories [14, 15, 17].

Application of the PVA cryogel incorporated with Fe_2_O_3_ NPs in in this study was shown to improve culture water characters. Water of this nano hydrogel was “very clean”. This treatment significantly reduced turbidity and ammonia content of water not only in comparison with the cadmium-treated culture, but also with the control culture. The reduction of ammonia in culture water suggests that the nano-gel also attracted nitrogenous remains. Together with the deposited material shown in the SEM image of the gel at the end of the culture, the results revealed an action of “sweeping and removing dirt” by the applied hydrogel from water in the fish farm.

The present study revealed obvious effects of cadmium on both nuclear and physiological levels of the tilapia *O. niloticus*. The results of the comet assay show that cadmium in culture water was able to cause significant increase of both tail length, tail DNA, and tail moment, indicating induction of primary DNA damage by cadmium presented as single-stranded breaks in the studied tilapia cells. These data agree with many previous reports in different tissues variable fish species [57–60]. Adsorption of cadmium by the applied nanogel prevented – to a great extent – this toxic effect and maintained the nuclear integrity.

In the present study, a significant decrease was observed in the total plasma protein content of *O. niloticus* upon exposure to cadmium. As well, total body protein was non-significantly decreased. This decrease may be a secondary action of the kidney damage caused by cadmium toxicity, which can cause protein loss and a consequent hypoproteinemia, or also secondary to the disturbance in protein synthesis on the cellular level or inhibition of blood protein synthesis in the liver [61, 62]. The decrease of plasma proteins has been reported to affect the transport and removal of toxic substances, including cadmium binding [63, 64].

Different environmental stressors increase carbohydrate metabolism with the consequent elevation of blood glucose levels [65, 66]. This elevation due to environmental stress conditions is commonly used as a key indicator for assessing fish health and stress status [67]. In the present study, cadmium exposure significantly caused hyperglycemia in *Oreochromis niloticus*. Many reasons were reported that cadmium exposure may increase blood glucose including interfering with glucose homeostasis, metalloenzymes, and lipid peroxidation, inhibition of insulin release and insulin receptor levels, activation of gluconeogenesis enzymes, excessive oxidative injuries, and alterations in DNA and membrane structures and functions [68, 69]. In agreement with the present results, many studies reported a significant increase in glucose level in different fish species after cadmium exposure, which is mediated by stress responses including the different reasons mentioned above [70–72].

In the same context, the results showed a significant increase of cortisol level. A concomitant elevation of both cortisol and glucose in response to cadmium intoxication was early reported in *Oreochromis mossambicus* [70] and other fish species [73]. Together with metallothionein, cortisol was considered the most sensitive index to stress. The significant lowering of both glucose and cortisol in the present result indicate that tilapia is exposed to less stress after treatment with the iron oxide nano gel.

Absorbed cadmium accumulates in different organs, including the kidney, liver, and gills, and the liver is always the first target for cadmium accumulation at high concentrations [74]. The hepatic transaminases ALT and AST are the most important liver function markers that secreted into the plasma upon tissue damage and dysfunction induced by toxicant exposure. In the present study, the plasma ALT and AST levels in *O. niloticus* were significantly increased upon exposure to cadmium. Their elevation indicates that cadmium exposure induces liver damage in tilapia, resulting in the leakage of ALT and AST into the bloodstream. These results agree with that of previous reports in different fish species, including tilapia [7, 75]. The kidney also is the second target of cadmium toxicity. The present study showed an increase of urea level, as a kidney function marker, upon cadmium exposure, compared to the control group. This urea increase in cadmium exposed fish may be attributed to the glomerular inefficiency and kidney dysfunction [75].

Hematological indices are indicators of health status in different fish types, since they reflect the physiological alterations after exposure to different stressors as pollutants, including heavy metals, and less oxygen supply. These parameters have different sensitivity to various environmental factors and chemicals, and the change of water quality. Hematological analyses in the present work were harmed by cadmium toxicity, an effect that was partially lessened by the nano gel treatment. White blood cell (WBC) count in cadmium group was found to be significantly less than the control, reflecting cadmium’s deleterious effect on the fish immunity. This decrease is mostly due to the great noticed lymphocytopenia. Fish immunosuppression and reduced disease resistance by cadmium toxicity was previously reported [76]. Regarding RBCs and hemoglobin, cadmium is also known to cause anemia through hemolysis, less cellular iron uptake, a decrease in cell viability, a decrease in red blood cell synthesis, and deficient erythropoietin production in different fish species [77], including tilapia [61]. In the present work, cadmium exposure significantly decreased the RBC count, hematocrit value and hemoglobin concentration in tilapia fish, suggesting that cadmium exposure can cause toxicity by targeting the hematological properties of tilapia. This hematopoietic impairment triggered by cadmium toxicity could be corrected by the nano treatment.

## Conclusion

We have developed a free-floating jellyfish-like nano-based structure composed of a PVA cryogel incorporated with green Fe_2_O_3_ NPs, biosynthesized by the metabolite of ***B***. *subtilis* bacteria at room temperature and in the presence of sunlight. This nano-based gel was proven to have antibacterial effect. Cadmium accumulation in the Nile tilapia *Oreochromis niloticus*, one of the most consumed fish in the world, showed a disruptive effect on the physiological processes and health of the fish, which threatens millions of tilapia-consuming humans. The prepared nano-based magnetic iron oxide gel could adsorb cadmium in tilapia aquaculture, improving the fish physiology and health. This was represented as maintaining the hepatic and renal cellular and nuclear integrity and functions, the main body and plasma composition, and improving the hematological and immunological parameters. The nano-based hydrogel has many advantages and presents an advanced technique in remediation of toxic heavy metals.

## Electronic supplementary material

Below is the link to the electronic supplementary material.

Supplementary Material 1

## Data Availability

All data and material are included in the manuscript.
